# Enlarged Spleen

**Published:** 1889-02

**Authors:** 


ENLARGED SPLEEN.—Dr. T. F. Bivins call for suggestions in the treatment of enlarged spleen as found in malarious sections.

We hope that some of our readers will respond. In the meanwhile we suggest to Dr. B. to try the following prescription:

| | | |
|---|---|---|
| ℞ | Iodide of ammonium, | 1 drachm, |
| | Fowler's sol. of arsenic, | ½ drachm, |
| | Tincture of colombo, | ½ ounce, |
| | Water, | 1½ ounces. |

Give teaspoonful three times a day and rub iodine oint. over the tumor.

